# Drugs quality supervision strategy of different distribution channels in pharmaceutical supply chain

**DOI:** 10.3389/fpubh.2022.954371

**Published:** 2022-10-11

**Authors:** Siyi Zhang, Lilong Zhu

**Affiliations:** ^1^School of Business, Shandong Normal University, Jinan, China; ^2^Quality Research Center, Shandong Normal University, Jinan, China

**Keywords:** drugs quality supervision, distribution channels, pharmaceutical supply chain, evolutionary games, simulation analysis

## Abstract

Aiming at the dual-channel pharmaceutical supply chain, which consists of two distribution channels, offline medical institutions, and online e-commerce platforms, and taking into account the impact of different strategic choices made by relevant stakeholders on the drugs quality of different distribution channels, this article constructs an evolutionary game model involving the participation of government regulator, pharmaceutical enterprises, medical institutions, and pharmaceutical e-commerce companies. The stable equilibrium points of each participant's strategic choices are solved; the stability of strategic combination is analyzed by *Lyapunov's first method*, and *MATLAB 2020b* is used for simulation to verify the influence of each decision variable on the strategic choice of different participants. The results show that, first, the purpose of punishment is to ensure the drugs quality in the pharmaceutical supply chain, but when the fine is too high, it will restrain the economic behavior of pharmaceutical enterprises, which is not conducive to the performance of social responsibilities by other relevant participants. Second, the probability that government regulator strictly supervises the pharmaceutical supply chain and the probability that pharmaceutical enterprises provide high-quality drugs are negatively related to their additional cost. Third, whether medical institutions and pharmaceutical e-commerce companies choose inspection is affected by multiple factors such as inspection cost, sales price, and sales cost. Furthermore, when the penalty for non-inspection of pharmaceutical e-commerce companies is greater than the threshold *F*_*m*0_, it can ensure that it chooses an inspection strategy. Finally, this article puts forward countermeasures and suggestions on the drugs quality supervision of different distribution channels in the pharmaceutical supply chain.

## Introduction

Nowadays, COVID-19 is still ongoing, and the entire pharmaceutical industry is constantly adapting to dramatic changes in the global economy and public health. The unique attributes of the drugs make their supply chains the most complex, and drugs supervision is gradually expanding to a global scale. Issues such as inconsistent drugs supervision standards, endless drugs problems, and lagging supervision concepts have become common challenges faced by all countries. In October 2020, Purdue Pharma admitted to inciting pharmacies and doctors to promote the company's “OxyContin” through bribery and other methods and agreed to pay a criminal fine of $3.544 billion and a civil penalty of $2.8 billion. In September 2020, the website of the French Competition Authority showed that three biopharmaceutical companies, Novartis Pharmaceuticals Group, Roche, and Genentech, were fined 445 million euros for market violations and alleged abuse of market dominance in marketing. In December 2020, the Japanese pharmaceutical company Kobayashi Chemical Co., Ltd. was ordered to suspend business for 116 days by the government for violating the “Drugs and Medical Devices Law” due to fraudulent behavior. In January 2021, China Simcere Pharmaceutical Group was fined 100.7 million CNY by the State Administration for Market Regulation according to law for its monopolistic behavior. In order to ensure the drugs quality and safety of the pharmaceutical supply chain, countries have formulated relevant strategies or plan to promote coordinated international supervision and global cooperation.

The pharmaceutical supply chain and the Internet are deeply integrated, the boundaries of enterprises are broken, and information technology forces the industrial chain to strengthen supply chain coordination. The application of Internet technology has produced a professional commodity trading network platform, using information technology to combine e-commerce with the traditional pharmaceutical distribution industry, forming a pharmaceutical supply chain with two distribution channels, such as online pharmaceutical e-commerce companies and offline medical institutions. However, the differentiation and information asymmetry between online and offline channels have exacerbated the dilemma of drugs quality and safety and restricted the drugs quality level of the pharmaceutical supply chain. How to realize the efficient coordination of the main bodies of the pharmaceutical supply chain under different distribution channels, ensure the drugs quality level of the pharmaceutical supply chain, and improve the supervision efficiency has become an urgent problem to be solved.

Therefore, this article considers the strategic choices of drugs quality supervision entities in the pharmaceutical supply chain under different distribution channel structures and constructs an evolutionary game model involving the participation of government regulator, pharmaceutical enterprises, medical institutions, and pharmaceutical e-commerce companies. By solving and analyzing the stability of strategic combination and the influence of each element on strategic choices, it aims to solve the following three problems. First, how does the strategic choice of pharmaceutical enterprises affect the behavior of government regulator, medical institutions, and pharmaceutical e-commerce companies? Second, how each decision variable affects the strategic choices of drugs quality supervision participants of different distribution channels in pharmaceutical supply chain? Third, how to promote government regulator, pharmaceutical enterprises, medical institutions, and pharmaceutical e-commerce companies actively perform their responsibilities and ensure the drugs quality level of the pharmaceutical supply chain?

The remaining parts of this article are arranged as follows. The second part combs and reviews the relevant literature; the third part makes hypotheses and constructs an evolutionary game model which involves government regulator, pharmaceutical enterprises, medical institutions, and pharmaceutical e-commerce companies; the fourth part analyzes the stability of the strategic choice of the four participants; the fifth part analyzes the stability of strategy combination according to the *Lyapunov's first method*; the sixth part is the simulation analysis with *MATLAB 2020b*; the seventh part discusses and outlines related suggestions; the last part suggests the conclusions.

## Relevant literature

### Pharmaceutical supply chain

The pharmaceutical supply chain covers many links such as raw material supply companies, pharmaceutical enterprises, terminal drugs sales agencies, and patients (1). The pharmaceutical supply chain is complex, and there are many drugs quality risk nodes (2), and there are problems such as non-standard drugs production testing and imperfect supervision mechanisms, which have become a research hotspot. Provide a competitive pharmaceutical supply chain under the product life cycle (3), supervise activities in the supply chain (4), and avoid monopoly (5) and collusion (6) among pharmaceutical supply chain entities, so as to better provide pharmaceutical retail services. Pharmaceutical products are perishable and improving the reliability, safety, and efficiency of the supply chain is the goal of the development and research of the pharmaceutical supply chain (7).

### Distribution channels

Inventory management and distribution network of pharmaceutical products is one of the most important issues in the medical field (8). Online and offline sales' channels in the pharmaceutical supply chain have their own unique advantages. Online channels are more convenient and faster than offline channels, which expands purchasing opportunities (9), but cannot provide accurate consultation services (10). The pharmaceutical retail industry adopts the O2O model, which has the risk of putting online and offline on the opposite side of the competition (11). The pursuit of profit maximization by pharmaceutical enterprises as suppliers and the existence of competition between online and offline retailers will affect the overall quality level of the pharmaceutical supply chain (12). The supply chain in which suppliers cooperate with retailers of different channels cannot achieve complete coordination and competition, and multi-channels bring greater challenges to the drugs quality supervision of the pharmaceutical supply chain (13).

### Drugs quality supervision

The quality supervision of the pharmaceutical supply chain determines the level of drugs safety (14). The intensity of government supervision should be gradually increased from the raw material suppliers (15), and the downstream entities should improve the testing level (16). By establishing a blockchain-based scenario application model and achieving a collaborative management mechanism (17), the purpose of shortening supply chain management time, improving efficiency, and meeting demand can be achieved. Government regulator should play a more active role (18) in further optimizing the decision-making process of pharmaceutical supply chain supervision (19), and giving economic penalties or government subsidies (20) can effectively promote pharmaceutical enterprises to provide high-quality drugs (21) and improve the quality of pharmaceutical supply chain (22).

To sum up, the existing literature mainly discusses the optimal decision-making of pharmaceutical supply chain members and how to improve the profit and efficiency of the supply chain. There is still a lack of research that simultaneously analyzes the strategic choice, mutual influence, and stability of strategic combination of the four participants: government regulator, pharmaceutical enterprises, medical institutions, and pharmaceutical e-commerce companies in drugs quality supervision.

Therefore, the research contributions of this article have the following three points. First, considering the strategic choices of government regulator, pharmaceutical enterprises, medical institutions, and pharmaceutical e-commerce companies in the drugs quality supervision of the pharmaceutical supply chain under different distribution channels, a game model involving four parties is constructed. Second, considering the influence of whether pharmaceutical enterprises provide high-quality drugs on the strategic choices of other participants, the evolutionary stable strategic combinations under different conditions are solved. Finally, the influence of the changes in different factors on the strategic choice of each participant is solved and analyzed, and the validity of the model is verified through the simulation analysis of *MATLAB 2020b*. Provide countermeasures and suggestions for the government regulator, pharmaceutical enterprises, medical institutions, and pharmaceutical e-commerce companies to participate in the drugs quality supervision of the pharmaceutical supply chain.

## Model hypotheses and construction

This article chooses the evolutionary game as the research method because it can explain the dynamic process of the evolution of each stakeholder's strategic choice and explain why this state has been reached and how to reach it.

**Figure 1:**
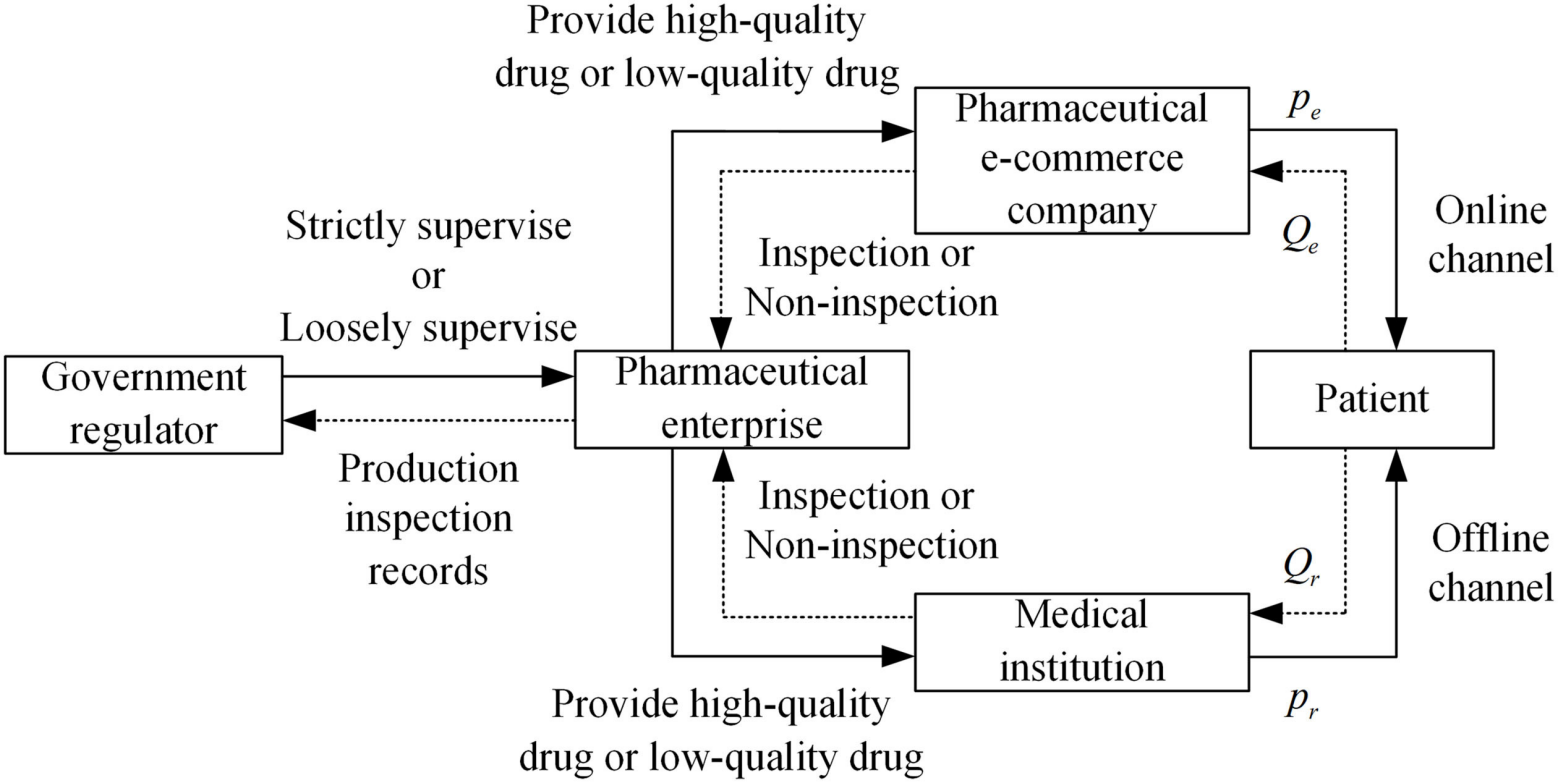
Drugs quality regulatory structure relationship. Figure is the structural relationship diagram that shows the relationship among government regulator, pharmaceutical enterprises, medical institutions, and pharmaceutical e-commerce companies in the drugs quality supervision of different distribution channels in pharmaceutical supply chain.

Therefore, considering a pharmaceutical dual-channel supply chain consisting of a pharmaceutical supplier, an offline medical institution, and an online e-commerce platform, this article constructs the drugs quality supervision structural relationship as shown in Figure 1.

### Model hypotheses

*H1* Select four participants: government regulator, pharmaceutical enterprises, medical institutions, and pharmaceutical e-commerce companies. In an evolutionary game, the four parties are all bounded rational participants, and the strategy choice evolves and stabilizes to the optimal strategy over time, and assume that the strategic choice space of the government regulator is *S*_1_ = (strictly supervise, loosely supervise); the strategic choice space of pharmaceutical enterprises is *S*_2_ = (provide high-quality drugs, provide low-quality drugs); the strategic choice space of medical institutions is *S*_3_ = (inspection, non-inspection); the strategic choice space of pharmaceutical e-commerce companies is *S*_4_ = (inspection, non-inspection).

*H2* The probability of government regulator choosing strict supervision is *x*(0 ≤ *x* ≤ 1), and the probability of choosing loose supervision is 1 − *x*. The probability of pharmaceutical enterprises choosing to provide high-quality drugs is *y*(0 ≤ *y* ≤ 1), and the probability of choosing to provide low-quality drugs is 1 − *y*. The probability of medical institutions choosing inspection is *z*(0 ≤ *z* ≤ 1), and the probability of choosing non-inspection is 1 − *z*. The probability of pharmaceutical e-commerce companies choosing inspection is *f*(0 ≤ *f* ≤ 1), and the probability of choosing non-inspection is 1 − *f*.

*H3* The utility obtained by patients from purchasing the drugs from offline medical institutions and online e-commerce platforms are: *V*_*r*_ = λ − *p*_*r*_. λ is the willingness of patients to buy drugs offline, evenly distributed on [0, *b*] *V*_*e*_ = ρλ − *p*_*e*_, ρ is the acceptance of online purchases by patients, and *p*_*r*_ and *p*_*e*_ are the retail prices of drugs, respectively. Considering the rationality of consumers, patients always choose the way of purchasing drugs based on the principle of maximizing utility, that is, *V*_max_ = max{*V*_*r*_, *V*_*e*_}. When $\frac{p_{r}-p_{e}}{1-\rho }\leq \lambda <b$, patients choose to buy drugs offline; when $\frac{p_{e}}{\rho }\leq \lambda <\frac{p_{r}-p_{e}}{1-\rho }$, they choose to buy drugs online. Therefore, the sales volume of medical institutions and pharmaceutical e-commerce companies are respectively: $Q_{r}=b-\frac{p_{r}-p_{e}}{1-\rho }$, $Q_{e}=\frac{\rho p_{r}-p_{e}}{\rho \left(1-\rho \right)}$.

*H4* The cost of strict supervision is *G*_*h*_, and the cost of loose supervision is *G*_*l*_(*G*_*h*_ > *G*_*l*_ > 0). When government regulator loosely supervises, they will be fined as *F*_g_.

*H5* The cost of providing high-quality drugs is *C*_*h*_, and the cost of providing low-quality drugs is *C*_*l*_(*C*_*h*_ > *C*_*l*_ > 0). ω is the unit wholesale price of pharmaceutical enterprises to medical institutions and pharmaceutical e-commerce companies, *T* is the unit cost of exchanging goods of pharmaceutical enterprises, ω > *T* > 0. The marketing cost of medical institutions is *C*_*r*_, the marketing cost of pharmaceutical e-commerce companies is *C*_*e*_, and the unit inspection cost of drugs is *C*_θ_. Enterprises that provide low-quality drugs will be fined *F*_*s*_ by government regulator. The penalty for non-inspection by medical institutions and pharmaceutical e-commerce companies is *F*_*m*_.

The parameters and descriptions of this article are shown in Table 1.

**Table 1:** Related parameter description.

**Parameter**	**Description**	**Parameter**	**Description**
*x*	Probability of government regulator choosing strict supervision	*F*_*g*_	Punishment for loose supervision of government regulator
*y*	Probability of pharmaceutical enterprises providing high-quality drugs	*G*_*h*_	The cost of strict supervision of government regulator
*z*	Probability of inspection of medical institutions	*G*_*l*_	The cost of loose supervision of government regulator
*f*	Probability of inspection of pharmaceutical e-commerce companies	ρ	The acceptance of online purchases by patients
*p*_*r*_, *p*_*e*_	Retail prices of medical institutions and pharmaceutical e-commerce companies	*T*	The unit cost of exchanging goods of pharmaceutical enterprises
*Q*_*r*_, *Q*_*e*_	The sales volume of medical institutions and pharmaceutical e-commerce companies	λ	The willingness of patients to buy drugs offline
*C*_θ_, ω	The unit inspection cost, the unit wholesale price	*C*_*h*_	The cost of pharmaceutical enterprises providing high-quality drugs
*C*_*r*_, *C*_*e*_	The marketing cost of medical institutions and pharmaceutical e-commerce companies	*C*_*l*_	The cost of pharmaceutical enterprises providing low-quality drugs
*F*_*m*_	The penalty for non-inspection by medical institutions and pharmaceutical e-commerce companies	*F*_*s*_	Fines for providing low-quality drugs of pharmaceutical enterprises

### Model construction

Based on the above hypotheses, this article constructs a drugs quality supervision game matrix in the pharmaceutical supply chain involving government regulator, pharmaceutical enterprises, medical institutions, and pharmaceutical e-commerce companies, as shown in Table 2.

**Table 2:** Four-party mixed strategy game matrix.

**Choice of strategy**		**Medical institutions**	**Government regulator**			
			**Strictly supervise** ***x***		**Loosely supervise** 1 − ***x***	
			**Pharmaceutical e-commerce companies**			
			**Inspection *f***	**Non-inspection 1 − *f***	**Inspection *f***	**Non-inspection 1 − *f***
Pharmaceutical enterprises	Provide high-quality drugs *y*	Inspection *z*	ω(*Q*_*r*_ + *Q*_*e*_) − *C*_*h*_ (*p*_*r*_ − ω − *C*_θ_)*Q*_*r*_ − *C*_*r*_ − *G*_*h*_ (*p*_*e*_ − ω − *C*_θ_)*Q*_*e*_ − *C*_*e*_	ω(*Q*_*r*_ + *Q*_*e*_) − *C*_*h*_ (*p*_*r*_ − ω − *C*_θ_)*Q*_*r*_ − *C*_*r*_ − *G*_*h*_ (*p*_*e*_ − ω)*Q*_*e*_ − *C*_*e*_	ω(*Q*_*r*_ + *Q*_*e*_) − *C*_*h*_ (*p*_*r*_ − ω − *C*_θ_)*Q*_*r*_ − *C*_*r*_ − *G*_*l*_ (*p*_*e*_ − ω − *C*_θ_)*Q*_*e*_ − *C*_*e*_	ω(*Q*_*r*_ + *Q*_*e*_) − *C*_*h*_ (*p*_*r*_ − ω − *C*_θ_)*Q*_*r*_ − *C*_*r*_ − *G*_*l*_ (*p*_*e*_ − ω)*Q*_*e*_ − *C*_*e*_
		Non-inspection 1 − *z*	ω(*Q*_*r*_ + *Q*_*e*_) − *C*_*h*_ (*p*_*r*_ − ω)*Q*_*r*_ − *C*_*r*_ − *G*_*h*_ (*p*_*e*_ − ω − *C*_θ_)*Q*_*e*_ − *C*_*e*_	ω(*Q*_*r*_ + *Q*_*e*_) − *C*_*h*_ (*p*_*r*_ − ω)*Q*_*r*_ − *C*_*r*_ − *G*_*h*_ (*p*_*e*_ − ω)*Q*_*e*_ − *C*_*e*_	ω(*Q*_*r*_ + *Q*_*e*_) − *C*_*h*_ (*p*_*r*_ − ω)*Q*_*r*_ − *C*_*r*_ − *G*_*l*_ (*p*_*e*_ − ω − *C*_θ_)*Q*_*e*_ − *C*_*e*_	ω(*Q*_*r*_ + *Q*_*e*_) − *C*_*h*_ (*p*_*r*_ − ω)*Q*_*r*_ − *C*_*r*_ − *G*_*l*_ (*p*_*e*_ − ω)*Q*_*e*_ − *C*_*e*_
	Provide low-quality drugs 1 − *y*	Inspection *z*	(ω − *T*)(*Q*_*r*_ + *Q*_*e*_) − *C*_*l*_ − *F*_*s*_ (*p*_*r*_ − ω − *C*_θ_)*Q*_*r*_ − *C*_*r*_ *F*_*s*_ − *G*_*h*_ (*p*_*e*_ − ω − *C*_θ_)*Q*_*e*_ − *C*_*e*_	(ω − *T*)(*Q*_*r*_ + *Q*_*e*_) − *C*_*l*_ − *F*_*s*_ (*p*_*r*_ − ω − *C*_θ_)*Q*_*r*_ − *C*_*r*_ *F*_*s*_ + *F*_*m*_ − *G*_*h*_ (*p*_*e*_ − ω)*Q*_*e*_ − *C*_*e*_ − *F*_*m*_	(ω − *T*)(*Q*_*r*_ + *Q*_*e*_) − *C*_*l*_ − *F*_*s*_ (*p*_*r*_ − ω − *C*_θ_)*Q*_*r*_ − *C*_*r*_ *F*_*s*_ − *F*_*g*_ − *G*_*l*_ (*p*_*e*_ − ω − *C*_θ_)*Q*_*e*_ − *C*_*e*_	(ω − *T*)(*Q*_*r*_ + *Q*_*e*_) − *C*_*l*_ − *F*_*s*_ (*p*_*r*_ − ω − *C*_θ_)*Q*_*r*_ − *C*_*r*_ *F*_*s*_ + *F*_*m*_ − *F*_*g*_ − *G*_*l*_ (*p*_*e*_ − ω)*Q*_*e*_ − *C*_*e*_ − *F*_*m*_
		Non-inspection 1 − *z*	(ω − *T*)(*Q*_*r*_ + *Q*_*e*_) − *C*_*l*_ − *F*_*s*_ (*p*_*r*_ − ω)*Q*_*r*_ − *C*_*r*_ − *F*_*m*_ *F*_*s*_ + *F*_*m*_ − *G*_*h*_ (*p*_*e*_ − ω − *C*_θ_)*Q*_*e*_ − *C*_*e*_	(ω − *T*)(*Q*_*r*_ + *Q*_*e*_) − *C*_*l*_ − *F*_*s*_ (*p*_*r*_ − ω)*Q*_*r*_ − *C*_*r*_ − *F*_*m*_ *F*_*s*_ + 2*F*_*m*_ − *G*_*h*_ (*p*_*e*_ − ω)*Q*_*e*_ − *C*_*e*_ − *F*_*m*_	(ω − *T*)(*Q*_*r*_ + *Q*_*e*_) − *C*_*l*_ − *F*_*s*_ (*p*_*r*_ − ω)*Q*_*r*_ − *C*_*r*_ − *F*_*m*_ *F*_*s*_ + *F*_*m*_ − *F*_*g*_ − *G*_*l*_ (*p*_*e*_ − ω − *C*_θ_)*Q*_*e*_ − *C*_*e*_	ω(*Q*_*r*_ + *Q*_*e*_) − *C*_*l*_ (*p*_*r*_ − ω)*Q*_*r*_ − *C*_*r*_ − *G*_*l*_ (*p*_*e*_ − ω)*Q*_*e*_ − *C*_*e*_

## Analysis of the strategic choice stability

### The government regulator's strategic choice stability

The replicated dynamic equation and the first derivative of the government regulator's strategic choice are:


(1) $$\begin{array}{l} E_{x}=\left(1-y\right)F_{s}-G_{h}+\left(1-y\right)\left(2-z-f\right)F_{m} \end{array}$$

(2) $$E_{1-x}=(1-y)(1-z)fF_{m}-G_{l}+(1-y)(z+f-zf)(F_{s}-F_{g})$$

(3) $$\begin{array}{l} F(x)=dx/dt=x(E_{x}-\overset{-}{E})=x(1-x)(E_{x}-E_{1-x})\\ \;=x(1-x)[G_{l}-G_{h}+(1-y)F_{s}+(1-y)(2-z\\ \;-2f+zf)F_{m}-(1-y)(2+f-zf)(F_{s}-F_{g})] \end{array}$$

(4) $$\begin{array}{l} F^{\prime }(x)=(1-2x)[G_{l}-G_{h}+(1-y)F_{s}+(1-y)(2-z-2f\\ \;+zf)F_{m}-(1-y)(2+f-zf)(F_{s}-F_{g})] \end{array}$$

According to the stability theorem of differential equations, if the probability of the government regulator choosing strict supervision is to be in a stable state, the following conditions must be met: *F*(*x*) = 0 and *F*′(*x*) < 0.

**Proposition 1** When *y* < *y*_0_, *z* < *z*_0_ or *f* < *f*_0_, the government regulator's stabilization strategy is “strict supervision.” When *y* > *y*_0_, *z* > *z*_0_, or *f* > *f*_0_, its stabilization strategy is “loose supervision.” When *y* = *y*_0_, *z* = *z*_0_, or *f* = *f*_0_, we are unable to determine its stabilization strategy.

***Proof*** Make *G*(*y, z, f*) = *G*_*l*_ − *G*_*h*_ + (1 − *y*)*F*_*s*_ + (1 − *y*)(2 − *z*−2*f* + *zf*)*F*_*m*_−(1−*y*)(2+*f* − *zf*)(*F*_*s*_−*F*_*g*_), when *G*(*y, z, f*) = 0, the thresholds $y_{0}=1-\frac{G_{h}-G_{l}}{F_{s}+\left(2-z-2f+zf\right)F_{m}-\left(2+f-zf\right)\left(F_{s}-F_{g}\right)}$, $z_{0}=\frac{G_{h}-G_{l}-\left(1-y\right)F_{s}-\left(1-y\right)\left(2-2f\right)F_{m}+\left(1-y\right)\left(2+f\right)\left(F_{s}-F_{g}\right)}{\left(1-y\right)\left[f\left(F_{s}-F_{g}\right)-\left(1-f\right)F_{m}\right]}$ and $f_{0}=\frac{G_{h}-G_{l}-(1-y)F_{s}-(1-y)(2-z)F_{m}+2(1-y)(F_{s}-F_{g})}{\left(1-y)[(z-2)F_{m}-(1-z)(F_{s}-F_{g}\right)}$ can be calculated. Because ∂*G*(*y, z, f*)/∂*y* < 0, ∂*G*(*y, z, f*)/∂*z* < 0, and ∂*G*(*y, z, f*)/∂*f* < 0, *G*(*y, z, f*) is a decreasing function of *y*, *z* and *f*. When *y* < *y*_0_, *z* < *z*_0_, or *f* < *f*_0_, *G*(*y, z, f*) > 0, $F^{\prime }\left(x\right){|}_{x=1}<0$ and *F*(*x*)|_*x*=1_ = 0 can be calculated, so *x* = 1 is stable. When *y* > *y*_0_, *z* > *z*_0_, or *f* > *f*_0_, *G*(*y, z, f*) < 0, $F^{\prime }\left(x\right){|}_{x=0}<0$ and *F*(*x*)|_*x*=0_ = 0 can be calculated, so *x* = 0 is stable. When *y* = *y*_0_, *z* = *z*_0_, or*f* = *f*_0_, *G*(*y, z, f*) = 0 and *F*′(*x*) = 0 can be calculated, we are unable to determine a stable strategy.

Proposition 1 shows that if the probability of pharmaceutical enterprises to provide high-quality drugs increases, the stabilization strategy of government regulator will change from strict supervision to loose supervision. If the inspection probability of medical institutions and pharmaceutical e-commerce companies is reduced, the stabilization strategy of government regulator will change from loose supervision to strict supervision. Therefore, in the drugs quality supervision of distribution channel in the pharmaceutical supply chain, when pharmaceutical enterprises voluntarily provide high-quality drugs, and the online and offline dual-channel entities take the initiative to undertake the inspection obligations, the government regulator realizes this and chooses loose supervision.

In summary, the response function of *x* is


(5) $$x=\{\begin{array}{ccc} 0 & if & y>1-\frac{G_{h}-G_{l}}{F_{s}+(2-z-2f+zf)F_{m}-(2+f-zf)(F_{s}-F_{g})}\\ \left(0,1\right) & if & y=1-\frac{G_{h}-G_{l}}{F_{s}+(2-z-2f+zf)F_{m}-(2+f-zf)(F_{s}-F_{g})}\\ 1 & if & y<1-\frac{G_{h}-G_{l}}{F_{s}+(2-z-2f+zf)F_{m}-(2+f-zf)(F_{s}-F_{g})} \end{array}$$

**Figure 2:**
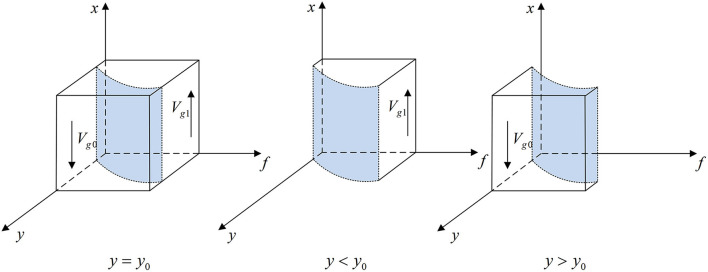
Phase diagram of the government regulator's strategic choice. Figure is the phase diagram that shows the evolutionary trend of government regulator's strategy obtained by calculating the response function of the probability of government regulator choosing the “strict supervision” strategy.

According to Proposition 1, the phase diagram of government regulator strategic choice is shown in Figure 2.

It can be seen from Figure 2 that the volume of *V*_*g*1_ is the probability that government regulator chooses the “strict supervision” strategy, and the volume of *V*_*g*0_ is the probability that it chooses the “loose supervision” strategy. And,


(6) $$\begin{array}{l} \begin{array}{l} V_{g1}=\int_{1}^{0}\int_{1}^{0}y_{0}dfdx=1-\frac{G_{h}-G_{l}}{\left(z-2)F_{m}-(1-z)(F_{s}-F_{g}\right)} \end{array}\\ \;\begin{array}{l} \ln\;\frac{F_{s}-(3-z)(F_{s}-F_{g})}{F_{s}+(2-z)F_{m}-2(F_{s}-F_{g})} \end{array} \end{array}$$

(7) $$\begin{array}{l} \begin{array}{l} V_{g0}=1-V_{g1}=\frac{G_{h}-G_{l}}{\left(z-2)F_{m}-(1-z)(F_{s}-F_{g}\right)} \end{array}\\ \;\begin{array}{l} \ln\;\frac{F_{s}-(3-z)(F_{s}-F_{g})}{F_{s}+(2-z)F_{m}-2(F_{s}-F_{g})} \end{array} \end{array}$$

**Corollary 1.1** The more additional costs government regulator pays for strict supervision, the less likely it will choose “strict supervision” strategy.

***Proof*** According to the probability *V*_*g*1_, the first-order partial derivatives of *G*_*h*_ − *G*_*l*_ can be calculated,


$$\begin{array}{l} \frac{\partial V_{g1}}{\partial (G_{h}-G_{l})}=-\frac{1}{\left(z-2)F_{m}-(1-z)(F_{s}-F_{g}\right)}\\ \;\ln\;\frac{F_{s}-(3-z)(F_{s}-F_{g})}{F_{s}+(2-z)F_{m}-2(F_{s}-F_{g})}<0 \end{array}$$

Corollary 1.1 shows that the probability of government regulator choosing “strict supervision” strategy is a decreasing function of *G*_*h*_ − *G*_*l*_. The greater the additional cost of strict supervision, the less likely it will conduct “strict supervision” in order to reduce costs.

**Corollary 1.2** The higher the *F*_*m*_, the greater the probability that the government regulator chooses the “loose supervision” strategy; the higher the *F*_*s*_, the lower the probability that it chooses the “loose supervision” strategy.

***Proof*** Make *M* = *F*_*s*_−(3−*z*)(*F*_*s*_−*F*_*g*_), *N* = *F*_*s*_+(2−*z*)*F*_*m*_−2(*F*_*s*_ − *F*_*g*_). According to the probability *V*_*g*0_, the first-order partial derivatives of *F*_*s*_ and *F*_*m*_ can be calculated,


$$\begin{array}{l} \frac{\partial V_{g0}}{\partial F_{s}}=-\frac{\left(G_{h}-G_{l})(1-z\right)}{{\left[(z-2)F_{m}-(1-z)(F_{s}-F_{g})\right]}^{2}}\ln\;\frac{N}{M}\\ \text{\#x000A0; }+\frac{\left(G_{h}-G_{l}\right)}{\left(z-2)F_{m}-(1-z)(F_{s}-F_{g}\right)}[\frac{z-2}{M}+\frac{1}{N}]<0\\ \frac{\partial V_{g0}}{\partial F_{m}}=\frac{\left(G_{h}-G_{l})(z-2\right)}{{\left[(z-2)F_{m}-(1-z)(F_{s}-F_{g})\right]}^{2}}\ln\;\frac{N}{M}\\ \;-\frac{\left(G_{h}-G_{l})(2-z\right)}{[(z-2)F_{m}-(1-z)(F_{s}-F_{g})]N}>0 \end{array}$$

Corollary 1.2 shows that the probability *V*_*g*0_ is a decreasing function of *F*_*s*_ and an increasing function of *F*_*m*_. The higher the fines imposed on medical institutions and pharmaceutical e-commerce companies, the more they will voluntarily conduct inspection, which can restrict the behavior of pharmaceutical enterprises. At this time, government regulator is more likely to choose loose supervision.

### Pharmaceutical enterprises' strategic choice stability

The replicated dynamic equation and the first derivative of pharmaceutical enterprises' strategic choice are:


(8) $$\begin{array}{l} E_{y}=\omega \left(Q_{r}+Q_{e}\right)-C_{h} \end{array}$$

(9) $$\begin{array}{l} E_{1-y}=(\omega -T)(Q_{r}+Q_{e})-C_{l}-F_{s}+[T(Q_{r}+Q_{e})\\ \;+F_{s}](1-z)(1-x)(1-f) \end{array}$$

(10) $$\begin{array}{l} F(y)=dy/dt=y(E_{y}-\overset{-}{E})=y(1-y)(E_{y}-E_{1-y})\\ \;=y(1-y)[T(b-p_{e}/\rho )+C_{l}+F_{s}-C_{h}-[T(b-p_{e}/\rho )\\ \;+F_{s}](1-z)(1-x)(1-f)] \end{array}$$

(11) $$\begin{array}{l} F^{\prime }(y)=(1-2y)[T(b-p_{e}/\rho )+C_{l}+F_{s}-C_{h}-[T(b-p_{e}/\rho )\\ \;+F_{s}](1-z)(1-x)(1-f)] \end{array}$$

According to the stability theorem of differential equations, if the probability of pharmaceutical enterprises choosing to provide high-quality drugs is to be in a stable state, the following conditions must be met: *F*(*y*) = 0 and *F*′(*y*) < 0.

**Proposition 2** When *x* > *x*_1_, *z* > *z*_1_ or *f* > *f*_1_, pharmaceutical enterprises' stabilization strategy is “providing high-quality drugs.” When *x* < *x*_1_, *z* < *z*_1_ or *f* < *f*_1_, their stabilization strategy is “providing low-quality drugs.” When *x* = *x*_1_, *z* = *z*_1_ or *f* = *f*_1_, we are unable to determine their stabilization strategy.

***Proof*** Make *K*(*x, z, f*) = *T*(*b* − *p*_*e*_/ρ) + *C*_*l*_ + *F*_*s*_ − *C*_*h*_ − [*T*(*b* − *p*_*e*_/ρ) + *F*_*s*_](1 − *z*)(1 − *x*)(1 − *f*), when *K*(*x, z, f*) = 0, the thresholds $x_{1}=1-\frac{T\left(b-p_{e}/\rho \right)+C_{l}+F_{s}-C_{h}}{\left[T\left(b-p_{e}/\rho \right)+F_{s}\right]\left(1-z\right)\left(1-f\right)}$, $z_{1}=1-\frac{T\left(b-p_{e}/\rho \right)+C_{l}+F_{s}-C_{h}}{\left[T\left(b-p_{e}/\rho \right)+F_{s}\right]\left(1-x\right)\left(1-f\right)}$, and $f_{1}=1-\frac{T\left(b-p_{e}/\rho \right)+C_{l}+F_{s}-C_{h}}{\left[T\left(b-p_{e}/\rho \right)+F_{s}\right]\left(1-z\right)\left(1-x\right)}$ can be calculated. Because ∂*K*(*x, z, f*)/∂*x* > 0, ∂*K*(*x, z, f*)/∂*z* > 0, and ∂*K*(*x, z, f*)/∂*f* > 0, *K*(*x, z, f*) is an increasing function of *x*, *z*, and *f*. When *x* > *x*_1_, *z* > *z*_1_, or *f* > *f*_1_, *K*(*x, z, f*) > 0, $F^{\prime }\left(y\right){|}_{y=1}<0$, and *F*(*y*)|_*y*=1_ = 0 can be calculated, so *y* = 1 is stable. When *x* < *x*_1_, *z* < *z*_1_, or *f* < *f*_1_, *K*(*x, z, f*) < 0, $F^{\prime }\left(y\right){|}_{y=0}<0$, and *F*(*y*)|_*y*=0_ = 0 can be calculated, so *y* = 0 is stable. When *x* = *x*_1_, *z* = *z*_1_, or *f* = *f*_1_, *K*(*x, z, f*) = 0 and *F*′(*y*) = 0 can be calculated, we are unable to determine a stable strategy.

Proposition 2 shows that with the increase of probability x, z, and f, the stable strategy of pharmaceutical enterprises will change from providing low-quality drugs to providing high-quality drugs. Therefore, the active provision of high-quality drugs by pharmaceutical enterprises requires the joint supervision of social entities.

In summary, the response function of *y* is


(12) $$y=\{\begin{array}{ccc} 0 & if & f<1-\frac{T(b-p_{e}/\rho )+C_{l}+F_{s}-C_{h}}{\left[T(b-p_{e}/\rho )+F_{s}](1-z)(1-x\right)}\\ \left(0,1\right) & if & f=1-\frac{T(b-p_{e}/\rho )+C_{l}+F_{s}-C_{h}}{\left[T(b-p_{e}/\rho )+F_{s}](1-z)(1-x\right)}\\ 1 & if & f>1-\frac{T(b-p_{e}/\rho )+C_{l}+F_{s}-C_{h}}{\left[T(b-p_{e}/\rho )+F_{s}](1-z)(1-x\right)} \end{array}$$

**Figure 3:**
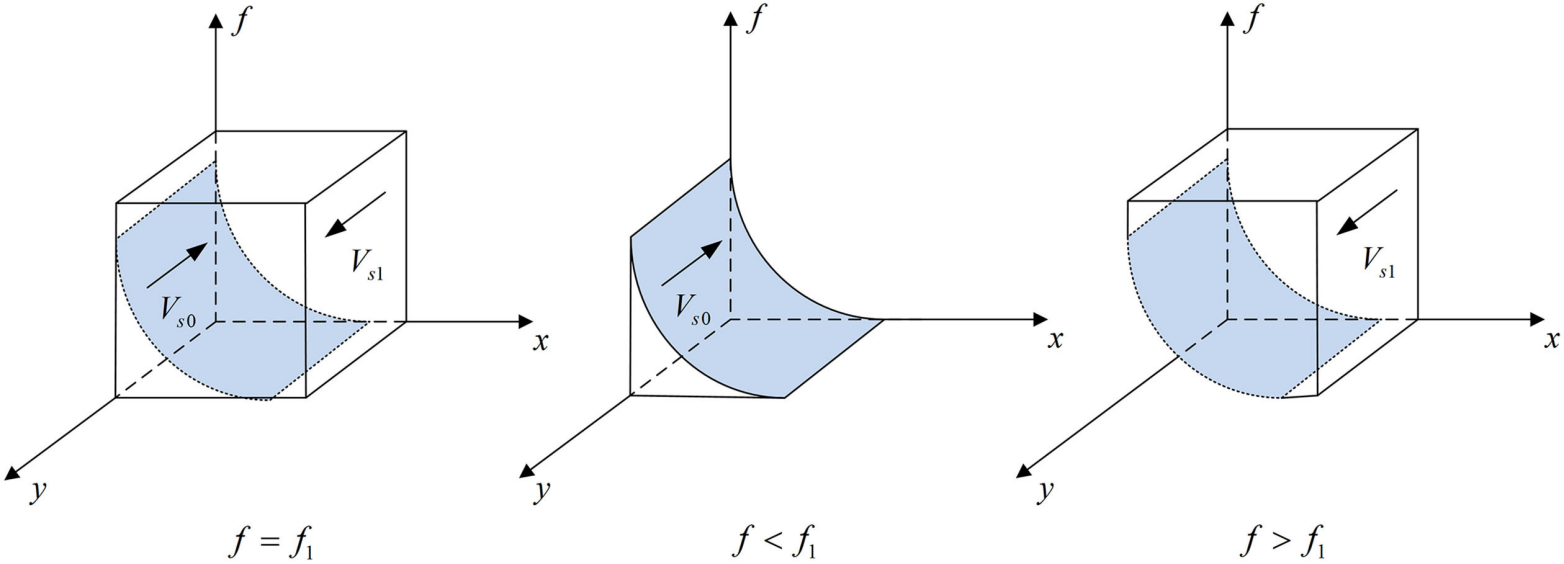
Phase diagram of pharmaceutical enterprises' strategic choice. Figure is the phase diagram that shows the evolutionary trend of pharmaceutical enterprises' strategy obtained by calculating the response function of the probability of pharmaceutical enterprises choosing the “providing high-quality drugs” strategy.

According to Proposition 2, the phase diagram of pharmaceutical enterprises' strategic choice is shown in Figure 3.

It can be seen from Figure 3 that the volume of *V*_*s*1_ is the probability that pharmaceutical enterprises choose the “providing high-quality drugs” strategy, and the volume of *V*_*s*0_ is the probability that they choose the “providing low-quality drugs” strategy. And,


(13) $$\begin{array}{l} V_{s0}=\int_{0}^{1}\int_{0}^{1-\frac{T(b-p_{e}/\rho )+C_{l}+F_{s}-C_{h}}{\left[T(b-p_{e}/\rho )+F_{s}](1-z\right)}}f_{1}dxdy\\ \;=1-\frac{T(b-p_{e}/\rho )+C_{l}+F_{s}-C_{h}}{\left[T(b-p_{e}/\rho )+F_{s}](1-z\right)}(1\\ \;-\ln\frac{T(b-p_{e}/\rho )+C_{l}+F_{s}-C_{h}}{\left[T(b-p_{e}/\rho )+F_{s}](1-z\right)}) \end{array}$$

(14) $$\begin{array}{l} V_{s1}=1-V_{s0}=\frac{T(b-p_{e}/\rho )+C_{l}+F_{s}-C_{h}}{\left[T(b-p_{e}/\rho )+F_{s}](1-z\right)}(1\\ \;-\ln\frac{T(b-p_{e}/\rho )+C_{l}+F_{s}-C_{h}}{\left[T(b-p_{e}/\rho )+F_{s}](1-z\right)}) \end{array}$$

**Corollary 2.1** The higher the additional cost for pharmaceutical enterprises to provide high-quality drugs, the less likely they are to provide high-quality drugs.

***Proof*** According to the probability *V*_*s*1_, the first-order partial derivatives of *C*_*h*_ − *C*_*l*_ can be calculated,


$$\begin{array}{l} \frac{\partial V_{s1}}{\partial (C_{h}-C_{l})}=-\frac{1}{\left[T(b-p_{e}/\rho )+F_{s}](1-z\right)}\\ \;\ln\;\frac{\left[T(b-p_{e}/\rho )+F_{s}](1-z\right)}{T(b-p_{e}/\rho )+C_{l}+F_{s}-C_{h}}<0 \end{array}$$

Corollary 2.1 shows that the probability *V*_*s*1_ is a decreasing function of *C*_*h*_ − *C*_*l*_. When the additional cost of providing high-quality drugs is greater, pharmaceutical enterprises are less willing to provide high-quality drugs for economic benefit.

**Corollary 2.2** The higher the unit cost of exchanging goods of pharmaceutical enterprises providing high-quality drugs, the lower the probability of providing low-quality drugs.

***Proof*** Make *A* = *T*(*b* − *p*_*e*_/ρ) + *C*_*l*_ + *F*_*s*_ − *C*_*h*_, *B* = [*T*(*b* − *p*_*e*_/ρ) + *F*_*s*_](1 − *z*). According to the probability *V*_*s*0_, the first-order partial derivatives of *T* can be calculated,


$$\begin{array}{l} \frac{\partial V_{s0}}{\partial T}=1-\frac{C_{h}-C_{l}}{B^{2}}(1-\ln\frac{A}{B})-(b\\ \;-\frac{p_{e}}{\rho })\frac{-z[T(b-p_{e}/\rho )+F_{s}]+C_{h}-C_{l}}{B^{2}}<0 \end{array}$$

Corollary 2.2 shows that the probability *V*_*s*0_ is a decreasing function of *T*. When the unit cost of exchanging goods is higher, the probability of pharmaceutical enterprises choosing to provide low-quality drugs will be smaller in order to ensure economic benefits.

### Medical institutions' strategic choice stability

The replicated dynamic equation and the first derivative of medical institutions' strategic choice are:


(15) $$\begin{array}{l} E_{z}=\left(p_{r}-\omega -C_{\theta }\right)Q_{r}-C_{r} \end{array}$$

(16) $$\begin{array}{l} E_{1-z}=\left(p_{r}-\omega \right)Q_{r}-C_{r}-\left(1-y\right)\left(x+f-xf\right)F_{m} \end{array}$$

(17) $$\begin{array}{l} F(z)=dz/dt=z(E_{z}-\overset{-}{E})=z(1-z)(E_{z}-E_{1-z})\\ \;=z(1-z)\{(1-y)(x+f-xf)F_{m}-[b-(p_{r}-p_{e})/(1\\ \;-\rho )]C_{\theta }\} \end{array}$$

(18) $$\begin{array}{l} F^{\prime }(z)=(1-2z)\{(1-y)(x+f-xf)F_{m}-[b-(p_{r}-p_{e})/(1\\ \;-\rho )]C_{\theta }\} \end{array}$$

According to the stability theorem of differential equations, if the probability of medical institutions choosing inspection is to be in a stable state, the following conditions must be met: *F*(*z*) = 0 and *F*′(*z*) < 0.

**Proposition 3** When *x* > *x*_2_, *y* < *y*_2_, or *f* > *f*_2_, medical institutions' stabilization strategy is “inspection.” When *x* < *x*_2_, *y* > *y*_2_, or *f* < *f*_2_, their stabilization strategy is “non-inspection.” When *x* = *x*_2_, *y* = *y*_2_, or *f* = *f*_2_, we are unable to determine their stabilization strategy.

***Proof*** Make *J*(*x, y, f*) = (1 − *y*)(*x* + *f* − *xf*)*F*_*m*_ − [*b* − (*p*_*r*_ − *p*_*e*_)/(1 − ρ)]*C*_θ_, when *J*(*x, y, f*) = 0, the thresholds $x_{2}=\frac{\left[b-\left(p_{r}-p_{e}\right)/\left(1-\rho \right)\right]C_{\theta }-\left(1-y\right)fF_{m}}{\left(1-y\right)\left(1-f\right)F_{m}}$, $y_{2}=1-\frac{\left[b-\left(p_{r}-p_{e}\right)/\left(1-\rho \right)\right]C_{\theta }}{\left(x+f-xf\right)F_{m}}$, and $f_{2}=\frac{\left[b-\left(p_{r}-p_{e}\right)/\left(1-\rho \right)\right]C_{\theta }-\left(1-y\right)xF_{m}}{\left(1-y\right)\left(1-x\right)F_{m}}$ can be calculated. Because ∂*J*(*x, y, f*)/∂*x*>0, ∂*J*(*x, y, f*)/∂*y* < 0, and ∂*J*(*x, y, f*)/∂*f*>0, *J*(*x, y, f*) is an increasing function of *x* and *f*, and a decreasing function of *y*. When *x* > *x*_2_, *y* < *y*_2_, or *f* > *f*_2_, *J*(*x, y, f*) > 0, $F^{\prime }\left(z\right){|}_{z=1}<0$, and *F*(*z*)|_*z*=1_ = 0 can be calculated, so *z* = 1 is stable. When *x* < *x*_2_, *y* > *y*_2_, or *f* < *f*_2_, *J*(*x, y, f*) < 0, $F^{\prime }\left(z\right){|}_{z=0}<0$, and *F*(*z*)|_*z*=0_ = 0 can be calculated, so *z* = 0 is stable. When *x* = *x*_2_, *y* = *y*_2_, or *f* = *f*_2_, *J*(*x, y, f*) = 0, and *F*′(*z*) = 0 can be calculated, we are unable to determine a stable strategy.

Proposition 3 shows that if the probability of strict supervision by government regulator and the probability of inspection of pharmaceutical e-commerce companies increase, or the probability of pharmaceutical enterprises to provide high-quality drugs decreases, the stability strategy of medical institutions will change from non-inspection to inspection. Therefore, the active behaviors of government regulator and pharmaceutical e-commerce companies will play a restrictive role in medical institutions.

In summary, the response function of *x* is


(19) $$z=\{\begin{array}{ccc} 0 & if & x<\frac{[b-(p_{r}-p_{e})/(1-\rho )]C_{\theta }-(1-y)fF_{m}}{(1-y)(1-f)F_{m}}\\ \left(0,1\right) & if & x=\frac{[b-(p_{r}-p_{e})/(1-\rho )]C_{\theta }-(1-y)fF_{m}}{(1-y)(1-f)F_{m}}\\ 1 & if & x>\frac{[b-(p_{r}-p_{e})/(1-\rho )]C_{\theta }-(1-y)fF_{m}}{(1-y)(1-f)F_{m}} \end{array}$$

**Figure 4:**
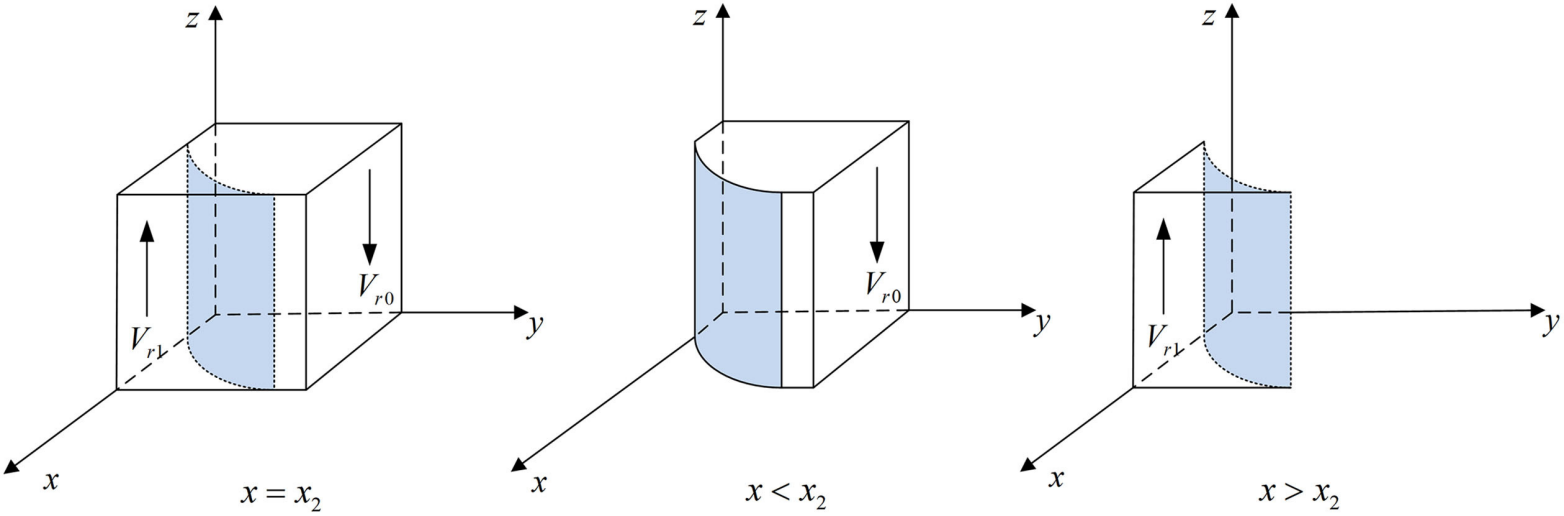
Phase diagram of medical institutions' strategic choice. Figure is the phase diagram that shows the evolutionary trend of medical institutions' strategy obtained by calculating the response function of the probability of medical institutions choosing the “inspection” strategy.

According to Proposition 3, the phase diagram of medical institutions strategic choice is shown in Figure 4.

It can be seen from Figure 4 that the volume of *V*_*r*1_ is the probability that the medical institutions choose the “inspection” strategy, and the volume of *V*_*r*0_ is the probability that they choose the “non-inspection” strategy. And,


(20) $$\begin{array}{l} V_{r0}=\int_{0}^{1}\int_{0}^{1-\frac{[b-(p_{r}-p_{e})/(1-\rho )]C_{\theta }}{F_{m}}}x_{2}dydz\\ \;=\frac{[b-(p_{r}-p_{e})/(1-\rho )]C_{\theta }}{(1-f)F_{m}}[f\\ \;-\ln\frac{[b-(p_{r}-p_{e})/(1-\rho )]C_{\theta }}{F_{m}}]-\frac{f}{1-f} \end{array}$$

(21) $$\begin{array}{l} V_{r1}=1-V_{r0}=1-\frac{[b-(p_{r}-p_{e})/(1-\rho )]C_{\theta }}{(1-f)F_{m}}[f\\ \;-\ln\frac{[b-(p_{r}-p_{e})/(1-\rho )]C_{\theta }}{F_{m}}]+\frac{f}{1-f} \end{array}$$

**Corollary 3.1** The probability of inspection by medical institutions is negatively related to the cost of inspection.

***Proof*** According to the probability *V*_*r*1_, the first-order partial derivatives of [*b*−(*p*_*r*_ − *p*_*e*_)/(1 − ρ)]*C*_θ_ can be calculated,


$$\begin{array}{l} \frac{\partial V_{r1}}{\partial [b-(p_{r}-p_{e})/(1-\rho )]C_{\theta }}=-\frac{1}{(1-f)F_{m}}[f+1\\ \;-\ln\frac{[b-(p_{r}-p_{e})/(1-\rho )]C_{\theta }}{F_{m}}]\\ \;<0 \end{array}$$

Corollary 3.1 shows that the higher the inspection cost of medical institutions, the lower the probability of choosing the “inspection” strategy.

**Corollary 3.2** The probability that medical institutions choose “non-inspection” is negatively related to their fine.

***Proof*** According to the probability *V*_*r*0_, the first-order partial derivatives of *F*_*m*_ can be calculated,


$$\begin{array}{l} \frac{\partial V_{r0}}{\partial F_{m}}=-\frac{[b-(p_{r}-p_{e})/(1-\rho )]C_{\theta }}{(1-f)F_{m}^{2}}[1-f\\ \;+\ln\frac{[b-(p_{r}-p_{e})/(1-\rho )]C_{\theta }}{F_{m}}]<0 \end{array}$$

Corollary 3.2 shows that when the price paid by medical institutions for non-inspection increases, the probability of choosing “non-inspecting” is lower.

### Pharmaceutical e-commerce companies' strategic choice stability

The replicated dynamic equation and the first derivative of pharmaceutical e-commerce companies' strategic choices are:


(22) $$\begin{array}{l} E_{f}=\left(p_{e}-\omega -C_{\theta }\right)Q_{e}-C_{e} \end{array}$$

(23) $$\begin{array}{l} E_{1-f}=\left(p_{e}-\omega \right)Q_{e}-C_{e}-\left(1-y\right)\left(x+z-xz\right)F_{m} \end{array}$$

(24) $$\begin{array}{l} F(f)=df/dt=f(E_{f}-\bar{E})=f(1-f)(E_{f}-E_{1-f})\\ \;=f(1-f)\{(1-y)(x+z-xz)F_{m}-[(\rho p_{r}\\ \;-p_{e})C_{\theta }]/[\rho (1-\rho )]\} \end{array}$$

(25) $$\begin{array}{l} F^{\prime }(f)=(1-2f)\{(1-y)(x+z-xz)F_{m}-[(\rho p_{r}\\ \;-p_{e})C_{\theta }]/[\rho (1-\rho )]\} \end{array}$$

According to the stability theorem of differential equations, if the probability of pharmaceutical e-commerce companies choosing inspection is to be in a stable state, the following conditions must be met: *F*(*f*) = 0 and *F*′(*f*) < 0.

**Proposition 4** When *x* > *x*_3_, *y* < *y*_3_, or *z* > *z*_3_, pharmaceutical e-commerce companies' stabilization strategy is “inspection.” When *x* < *x*_3_, *y* > *y*_3_, or *z* < *z*_3_, their stabilization strategy is “non-inspection.” When *x* = *x*_3_, *y* = *y*_3_, or *z* = *z*_3_, we are unable to determine their stabilization strategy.

***Proof*** Make *H*(*x, y, z*) = (1 − *y*)(*x* + *z* − *xz*)*F*_*m*_ − [(ρ*p*_*r*_ − *p*_*e*_)*C*_θ_]/[ρ(1 − ρ)], when *H*(*x, y, z*) = 0, the thresholds $x_{3}=\frac{\left[\left(\rho p_{r}-p_{e}\right)C_{\theta }\right]/\left[\rho \left(1-\rho \right)\right]-\left(1-y\right)zF_{m}}{\left(1-y\right)\left(1-z\right)F_{m}}$, $y_{3}=1-\frac{\left[\left(\rho p_{r}-p_{e}\right)C_{\theta }\right]/\left[\rho \left(1-\rho \right)\right]}{\left(x+z-xz\right)F_{m}}$, and $z_{3}=\frac{\left[\left(\rho p_{r}-p_{e}\right)C_{\theta }\right]/\left[\rho \left(1-\rho \right)\right]-\left(1-y\right)xF_{m}}{\left(1-y\right)\left(1-x\right)F_{m}}$ can be calculated. Because ∂*H*(*x, y, z*)/∂*x* > 0, ∂*H*(*x, y, z*)/∂*y* < 0, and ∂*H*(*x, y, z*)/∂*z* > 0, *H*(*x, y, z*) is an increasing function of *x* and *z*, and a decreasing function of *y*. When *x* > *x*_3_, *y* < *y*_3_ or *z* > *z*_3_, *H*(*x, y, z*) > 0, $F^{\prime }\left(f\right){|}_{f=1}<0$ and *F*(*f*)|_*f*=1_ = 0 can be calculated, so *f* = 1 is stable. When *x* < *x*_3_, *y* > *y*_3_, or *z* < *z*_3_, *H*(*x, y, z*) < 0, $F^{\prime }\left(f\right){|}_{f=0}<0$ and *F*(*f*)|_*f*=0_ = 0 can be calculated, so *f* = 0 is stable. When *x* = *x*_3_, *y* = *y*_3_, or *z* = *z*_3_, *H*(*x, y, z*) = 0 and *F*′(*f*) = 0 can be calculated, we are unable to determine a stable strategy.

Proposition 4 shows that if the probability of strict supervision increases, the probability of inspection by medical institutions increases, or the probability of pharmaceutical enterprises providing high-quality drugs decreases, the stable strategy of pharmaceutical e-commerce companies will change from non-inspection to inspection.

In summary, the response function of *f* is


(26) $$f=\{\begin{array}{ccc} 0 & if & x<\frac{[(\rho p_{r}-p_{e})C_{\theta }]/[\rho (1-\rho )]-(1-y)zF_{m}}{(1-y)(1-z)F_{m}}\\ \left(0,1\right) & if & x=\frac{[(\rho p_{r}-p_{e})C_{\theta }]/[\rho (1-\rho )]-(1-y)zF_{m}}{(1-y)(1-z)F_{m}}\\ 1 & if & x>\frac{[(\rho p_{r}-p_{e})C_{\theta }]/[\rho (1-\rho )]-(1-y)zF_{m}}{(1-y)(1-z)F_{m}} \end{array}$$

**Figure 5:**
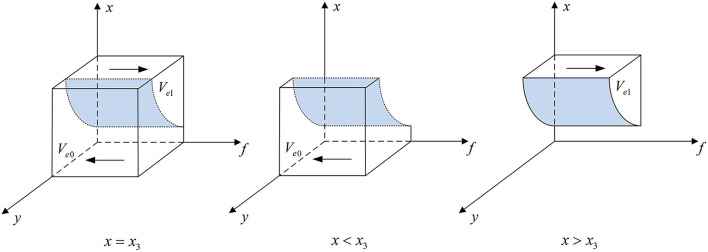
Phase diagram of pharmaceutical e-commerce companies' strategic choice. Figure is the phase diagram that shows the evolutionary trend of pharmaceutical e-commerce companies' strategy obtained by calculating the response function of the probability of pharmaceutical e-commerce companies choosing the “inspection” strategy.

According to Proposition 4, the phase diagram of pharmaceutical e-commerce companies' strategic choices is shown in Figure 5.

It can be seen from Figure 5 that the volume of *V*_*e*1_ is the probability that pharmaceutical e-commerce companies choose the “inspection” strategy, and the volume of *V*_*e*0_ is the probability that they choose the “non-inspection” strategy. And,


(27) $$\begin{array}{l} V_{e0}=\int_{0}^{1}\int_{0}^{1-\frac{(\rho p_{r}-p_{e})C_{\theta }}{\rho (1-\rho )F_{m}}}x_{3}dydf\\ \;=\frac{(\rho p_{r}-p_{e})C_{\theta }}{\rho (1-\rho )(1-z)F_{m}}[z-\ln\frac{(\rho p_{r}-p_{e})C_{\theta }}{\rho (1-\rho )F_{m}}]\\ \;-\frac{z}{1-z} \end{array}$$

(28) $$\begin{array}{l} V_{e1}=1-V_{e0}=1-\frac{(\rho p_{r}-p_{e})C_{\theta }}{\rho (1-\rho )(1-z)F_{m}}[z\\ \;-\ln\frac{(\rho p_{r}-p_{e})C_{\theta }}{\rho (1-\rho )F_{m}}]+\frac{z}{1-z} \end{array}$$

**Corollary 4.1** The inspection probability of pharmaceutical e-commerce companies is positively correlated with online drugs retail price.

***Proof*** According to the probability *V*_*e*1_, the first-order partial derivatives of *p*_*e*_ can be calculated,


$$\begin{array}{l} \frac{\partial V_{e1}}{\partial p_{e}}=\frac{C_{\theta }}{\rho \left(1-\rho \right)\left(1-z\right)F_{m}}\left[z-1+\ln\;\frac{\rho \left(1-\rho \right)F_{m}}{\left(\rho p_{r}-p_{e}\right)C_{\theta }}\right]>0 \end{array}$$

Corollary 4.1 shows that with the increase in online drugs retail price, pharmaceutical e-commerce companies are more inclined to conduct inspections on drugs quality.

**Corollary 4.2** When *F*_*m*_ > *F*_*m*0_, pharmaceutical e-commerce companies will choose the “inspection” strategy. When *F*_*m*_ < *F*_*m*0_, they will choose the “non-inspection” strategy. The threshold is $F_{m0}=\frac{\left(\rho p_{r}-p_{e}\right)C_{\theta }}{\rho \left(1-\rho \right)\left(1-y\right)\left(x+z-xz\right)}$.

***Proof*** According to Proposition 4, when *H*(*x, y, z*) = 0, $F_{m0}=\frac{\left(\rho p_{r}-p_{e}\right)C_{\theta }}{\rho \left(1-\rho \right)\left(1-y\right)\left(x+z-xz\right)}$ can be calculated. Because ∂*H*(*x, y, z*)/∂*F*_*m*_ > 0, *H*(*x, y, z*) is an increasing function of *F*_*m*_. When *F*_*m*_ > *F*_*m*0_, *H*(*x, y, z*) > 0, $F^{\prime }\left(f\right){|}_{f=1}<0$ and *F*(*f*)|_*f*=1_ = 0 can be calculated. When *F*_*m*_ < *F*_*m*0_, *H*(*x, y, z*) < 0, $F^{\prime }\left(f\right){|}_{f=0}<0$, and *F*(*f*)|_*f*=0_ = 0 can be calculated.

Corollary 4.2 shows that, when *F*_*m*_ > *F*_*m*0_, it can ensure that pharmaceutical e-commerce companies choose the “inspection” strategy. Therefore, it is necessary to increase the degree of punishment for pharmaceutical e-commerce companies that do not inspect to restrain their behavior.

## Stability analysis of strategic combination

In the replication dynamic system of the quadrilateral game between government regulator, pharmaceutical enterprises, medical institutions, and pharmaceutical e-commerce companies, the stability of the strategic combination can be judged according to the *Lyapunov's first method*. If all the eigenvalues of the *Jacobian matrix* are negative, the equilibrium point is the progressive evolutionary stable strategy (ESS). If at least one of the eigenvalues is positive, the equilibrium point is unstable. If the eigenvalues are all negative except zero, the equilibrium point is in a critical state and the stability is uncertain. This article analyzes the stability of the 16 pure strategy Nash equilibrium points, the *Jacobian matrix* of the replication dynamic system is,


$$\begin{array}{l} J=\left[\begin{array}{cccc} \partial F\left(x\right)/\partial x & \partial F\left(x\right)/\partial y & \partial F\left(x\right)/\partial z & \partial F\left(x\right)/\partial f\\ \partial F\left(y\right)/\partial x & \partial F\left(y\right)/\partial y & \partial F\left(y\right)/\partial z & \partial F\left(y\right)/\partial f\\ \partial F\left(z\right)/\partial x & \partial F\left(z\right)/\partial y & \partial F\left(z\right)/\partial z & \partial F\left(z\right)/\partial f\\ \partial F\left(f\right)/\partial x & \partial F\left(f\right)/\partial y & \partial F\left(f\right)/\partial z & \partial F\left(f\right)/\partial f \end{array}\right] \end{array}$$

### When pharmaceutical enterprises provide high-quality drugs

If the stability strategy of pharmaceutical enterprises is “providing high-quality drugs,” that is, when condition *T*(*b* − *p*_*e*_/ρ) > *C*_*h*_−*C*_*l*_−*F*_*s*_ is satisfied, the asymptotic stability analysis of the equilibrium point of the replicated dynamic system is shown in Table 3.

**Table 3:** Asymptotic stability of the equilibrium point in providing high-quality drugs.

**Equilibrium point**	**Eigenvalues λ_1_, λ_2_, λ_3_, λ_4_**	**Sign**	**Stability**
(1, 1, 1, 1)	*G*_*h*_ − *G*_*l*_, −[*T*(*b* − *p*_*e*_/ρ) + *C*_*l*_ + *F*_*s*_ − *C*_*h*_], [*b* − (*p*_*r*_ − *p*_*e*_)/(1 − ρ)]*C*_θ_, (ρ*p*_*r*_ − *p*_*e*_)*C*_θ_/ρ(1 − ρ)	(+, −, +, +)	Unstable
(1, 1, 1, 0)	*G*_*h*_ − *G*_*l*_, −[*T*(*b* − *p*_*e*_/ρ) + *C*_*l*_ + *F*_*s*_ − *C*_*h*_], [*b* − (*p*_*r*_ − *p*_*e*_)/(1 − ρ)]*C*_θ_, −(ρ*p*_*r*_ − *p*_*e*_)*C*_θ_/ρ(1 − ρ)	(+, −, +, −)	Unstable
(1, 1, 0, 1)	*G*_*h*_ − *G*_*l*_, −[*T*(*b* − *p*_*e*_/ρ) + *C*_*l*_ + *F*_*s*_ − *C*_*h*_], − ([*b* − (*p*_*r*_ − *p*_*e*_)/(1 − ρ)]*C*_θ_, (ρ*p*_*r*_ − *p*_*e*_)*C*_θ_/ρ(1 − ρ)	(+, −, −, +)	Unstable
(1, 1, 0, 0)	*G*_*h*_ − *G*_*l*_, −[*T*(*b* − *p*_*e*_/ρ) + *C*_*l*_ + *F*_*s*_ − *C*_*h*_], −[*b* − (*p*_*r*_ − *p*_*e*_)/(1 − ρ)]*C*_θ_, −(ρ*p*_*r*_ − *p*_*e*_)*C*_θ_/ρ(1 − ρ)	(+, −, −, −)	Unstable
(0, 1, 0, 0)	*G*_*l*_ − *G*_*h*_, *C*_*h*_ − *C*_*l*_, −[*b* − (*p*_*r*_ − *p*_*e*_)/(1 − ρ)]*C*_θ_, −(ρ*p*_*r*_ − *p*_*e*_)*C*_θ_/ρ(1 − ρ)	(−, +, −, −)	Unstable
(0, 1, 0, 1)	*G*_*l*_ − *G*_*h*_, −[*T*(*b* − *p*_*e*_/ρ) + *C*_*l*_ + *F*_*s*_ − *C*_*h*_], −[*b* − (*p*_*r*_ − *p*_*e*_)/(1 − ρ)]*C*_θ_, (ρ*p*_*r*_ − *p*_*e*_)*C*_θ_/ρ(1 − ρ)	(−, −, −, +)	Unstable
(0, 1, 1, 0)	*G*_*l*_ − *G*_*h*_, −[*T*(*b* − *p*_*e*_/ρ) + *C*_*l*_ + *F*_*s*_ − *C*_*h*_], [*b* − (*p*_*r*_ − *p*_*e*_)/(1 − ρ)]*C*_θ_, −(ρ*p*_*r*_ − *p*_*e*_)*C*_θ_/ρ(1 − ρ)	(−, −, +, −)	Unstable
(0, 1, 1, 1)	*G*_*l*_ − *G*_*h*_, −[*T*(*b* − *p*_*e*_/ρ) + *C*_*l*_ + *F*_*s*_ − *C*_*h*_], [*b* − (*p*_*r*_ − *p*_*e*_)/(1 − ρ)]*C*_θ_, (ρ*p*_*r*_ − *p*_*e*_)*C*_θ_/ρ(1 − ρ)	(−, −, −, −)	ESS

It can be seen from Table 3 that if pharmaceutical enterprises provide high-quality drugs, the equilibrium point (0, 1, 1, 1) of the replicated dynamic system is ESS.

The most ideal situation for government regulator is pharmaceutical enterprises providing high-quality drugs, inspected by medical institutions, and pharmaceutical e-commerce companies are. At this time, in order to save financial expenditures and maximize social interests, the government regulator will carry out loose supervision.

### When pharmaceutical enterprises provide low-quality drugs

If the stability strategy of pharmaceutical enterprises is “providing low-quality drugs,” that is, when condition *T*(*b* − *p*_*e*_/ρ) < *C*_*h*_−*C*_*l*_−*F*_*s*_ is satisfied, the asymptotic stability analysis of the equilibrium point of the replicated dynamic system is shown in Table 4.

**Table 4:** Asymptotic stability of the equilibrium point in providing low-quality drugs.

**Equilibrium point**	**Eigenvalues λ_1_, λ_2_, λ_3_, λ_4_**	**Sign**	**Stability**
(1, 0, 1, 0)	*G*_*h*_ − *G*_*l*_ + *F*_*s*_ − *F*_*m*_ − 2*F*_*g*_, *T*(*b* − *p*_*e*_/ρ) + *C*_*l*_ + *F*_*s*_ − *C*_*h*_, [*b* − (*p*_*r*_ − *p*_*e*_)/(1 − ρ)]*C*_θ_ − *F*_*m*_, *F*_*m*_ − [(ρ*p*_*r*_ − *p*_*e*_)*C*_θ_/ρ(1 − ρ)]	(×, −, +, −)	Unstable
(1, 0, 1, 1)	*G*_*h*_ − *G*_*l*_ + *F*_*s*_ − 2*F*_*g*_, *T*(*b* − *p*_*e*_/ρ) + *C*_*l*_ + *F*_*s*_ − *C*_*h*_, [*b* − (*p*_*r*_ − *p*_*e*_)/(1 − ρ)]*C*_θ_ − *F*_*m*_, [(ρ*p*_*r*_ − *p*_*e*_)*C*_θ_/ρ(1 − ρ)] − *F*_*m*_	(×, −, +, +)	Unstable
(1, 0, 0, 0)	*G*_*h*_ − *G*_*l*_ + *F*_*s*_ − 2*F*_*m*_ − 2*F*_*g*_, *T*(*b* − *p*_*e*_/ρ) + *C*_*l*_ + *F*_*s*_ − *C*_*h*_, *F*_*m*_ − [*b* − (*p*_*r*_ − *p*_*e*_)/(1 − ρ)]*C*_θ_, *F*_*m*_ − [(ρ*p*_*r*_ − *p*_*e*_)*C*_θ_/ρ(1 − ρ)]	(×, −, −, −)	When condition ① is satisfied, it is ESS
(1, 0, 0, 1)	*G*_*h*_ − *G*_*l*_ + 2*F*_*s*_ − 3*F*_*g*_, *T*(*b* − *p*_*e*_/ρ) + *C*_*l*_ + *F*_*s*_ − *C*_*h*_, *F*_*m*_ − [*b* − (*p*_*r*_ − *p*_*e*_)/(1 − ρ)]*C*_θ_, [(ρ*p*_*r*_ − *p*_*e*_)*C*_θ_/ρ(1 − ρ)] − *F*_*m*_	(×, −, −, +)	Unstable
(0, 0, 0, 0)	*G*_*l*_ − *G*_*h*_ − *F*_*s*_ + 2*F*_*m*_ + 2*F*_*g*_, *C*_*l*_ − *C*_*h*_, − [*b* − (*p*_*r*_ − *p*_*e*_)/(1 − ρ)]*C*_θ_, − (ρ*p*_*r*_ − *p*_*e*_)*C*_θ_/ρ(1 − ρ)	(×, −, −, −)	When condition ② is satisfied, it is ESS
(0, 0, 0, 1)	*G*_*l*_ − *G*_*h*_ − 2*F*_*s*_ + 3*F*_*g*_, *T*(*b* − *p*_*e*_/ρ) + *C*_*l*_ + *F*_*s*_ − *C*_*h*_, *F*_*m*_ − [*b* − (*p*_*r*_ − *p*_*e*_)/(1 − ρ)]*C*_θ_, (ρ*p*_*r*_ − *p*_*e*_)*C*_θ_/ρ(1 − ρ)	(×, −, −, +)	Unstable
(0, 0, 1, 0)	*G*_*l*_ − *G*_*h*_ − *F*_*s*_ + *F*_*m*_ + 2*F*_*g*_, *T*(*b* − *p*_*e*_/ρ) + *C*_*l*_ + *F*_*s*_ − *C*_*h*_, [*b* − (*p*_*r*_ − *p*_*e*_)/(1 − ρ)]*C*_θ_, *F*_*m*_ − [(ρ*p*_*r*_ − *p*_*e*_)*C*_θ_/ρ(1 − ρ)]	(×, −, +, −)	Unstable
(0, 0, 1, 1)	*G*_*l*_ − *G*_*h*_ − *F*_*s*_ + 2*F*_*g*_, *T*(*b* − *p*_*e*_/ρ) + *C*_*l*_ + *F*_*s*_ − *C*_*h*_, [*b* − (*p*_*r*_ − *p*_*e*_)/(1 − ρ)]*C*_θ_ − *F*_*m*_, [(ρ*p*_*r*_ − *p*_*e*_)*C*_θ_/ρ(1 − ρ)] − *F*_*m*_	(×, −, +, +)	Unstable

× indicates that the sign is uncertain. If the conditions ① and ② are met, they are, respectively, stable points.

Condition ①: *G*_*h*_ − *G*_*l*_ < 2*F*_*m*_ + 2*F*_*g*_ − *F*_*s*_; Condition ②: 2*F*_*m*_ + 2*F*_*g*_ − *F*_*s*_ < *G*_*h*_ − *G*_*l*_.

It can be seen from Table 4 that if condition is satisfied, the equilibrium point (1, 0, 0, 0) of the replication dynamic system is ESS. When condition is satisfied, the equilibrium point (0, 0, 0, 0) of the replication dynamic system is ESS.

The above two states of stable equilibrium point are not ideal. Strict supervision by government regulator is to play a deterrent role. The drugs quality supervision of the pharmaceutical supply chain requires the joint efforts of stakeholders and their respective social responsibilities in order to create a good business environment for pharmaceutical enterprises.

## Simulation analysis

Based on the above theoretical analysis, *MATLAB 2020b* is used to conduct an intuitive observation of numerical simulation, so as to obtain more enlightenment on the drugs quality supervision of distribution channels in the pharmaceutical supply chain. Combined with the actual situation and relevant literature content, the parameters are set as: *G*_*h*_ − *G*_*l*_ = 7, *C*_*h*_ − *C*_*l*_ = 7, *F*_*g*_ = 3, *F*_*s*_ = 4, *F*_*m*_ = 3, *C*_θ_ = 2, *C*_*e*_ = 2, *C*_*r*_ = 3, *p*_*e*_ = 3, *p*_*r*_ = 4, ω = 2, *b* = 5, and ρ = 0.5, *T* = 1.

### Impact of penalty

**Figure 6:**
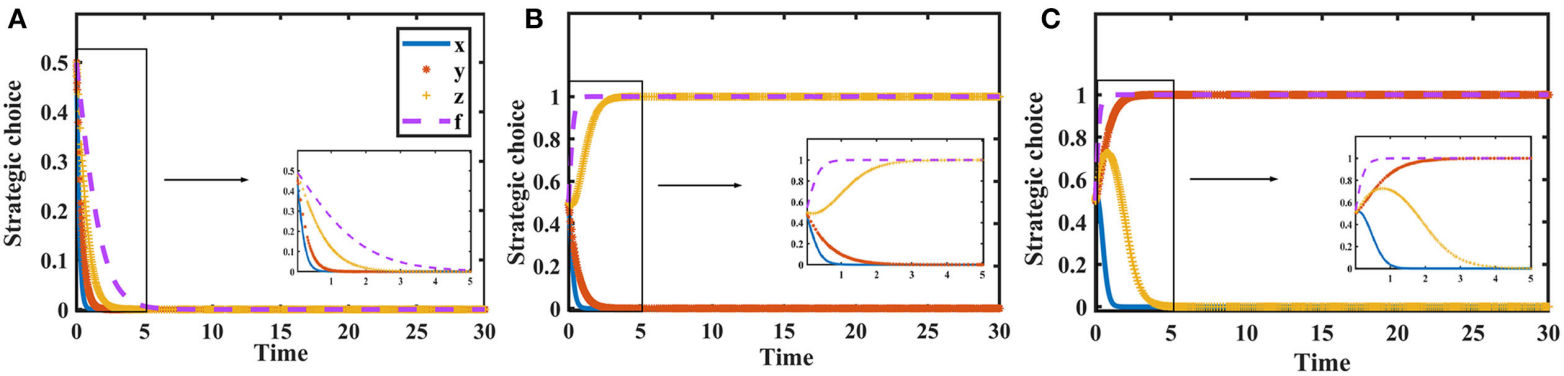
Impact of penalties on the evolution of each player's strategy. Figure is the simulation diagram that shows the influence of the penalties on the strategic choices of government regulator, pharmaceutical enterprises, medical institutions, and pharmaceutical e-commerce companies. **(A)** When *F*_*g*_ = 0, *F*_*s*_ = 0, *F*_*m*_ = 0; **(B)** when *F*_*g*_ = 4, *F*_*s*_ = 6, *F*_*m*_ = 4; **(C)** when *F*_*g*_ = 10, *F*_*s*_ = 10, *F*_*m*_ = 10.

Suppose that the penalty for loose supervision by the government regulator is *F*_*g*_ = {0, 4, 10}, the penalty for pharmaceutical enterprises for providing low-quality medicines is *F*_*s*_ = {0, 6, 10}, and the penalty for non-inspection by medical institutions and pharmaceutical e-commerce companies is *F*_*m*_ = {0, 4, 10}. The evolution process and results of players' strategy in the quartet game are shown in Figure 6.

Figure 6 shows that when the fines are 0, the government regulator will loosely supervise, pharmaceutical enterprises provide low-quality drugs, and medical institutions and pharmaceutical e-commerce companies do not inspect. As the fines increase, the stable strategies of medical institutions and pharmaceutical e-commerce companies are changed to inspection. When the fines are further increased, the cost of providing low-quality drugs is too high for pharmaceutical enterprises, and they will choose to provide high-quality drugs, but at this time other entities realize this and will not conduct supervision and inspection in order to save costs.

### Impact of cost of exchanging goods

**Figure 7:**
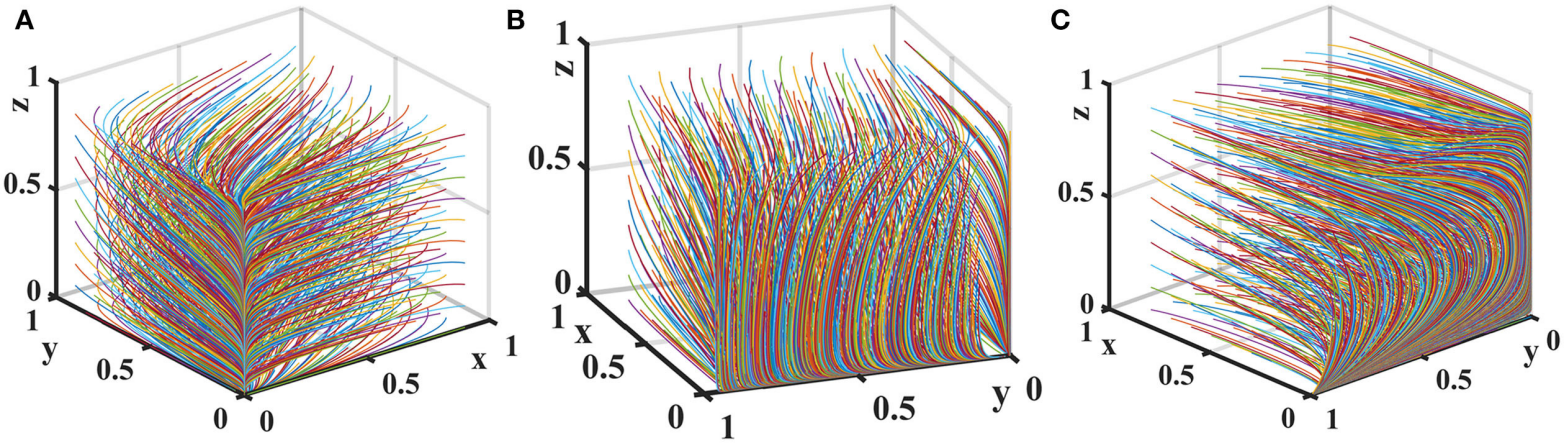
Impact of *T* on the evolution of each player's strategy. Figure is the simulation diagram that shows the influence of the unit cost of exchanging goods of pharmaceutical enterprises on the strategic choices of government regulator, pharmaceutical enterprises, and medical institutions. **(A)** When *T* = 0; **(B)** when *T* = 2; **(C)** when *T* = 4.

Let the probability that pharmaceutical e-commerce companies choose inspection is *f* = 0.5. Suppose the unit cost of exchanging goods is *T* = {0, 2, 4}. The tripartite game evolutionary process and results of government regulator, pharmaceutical enterprises, and medical institutions are shown in Figure 7.

Figure 7 shows that when the unit cost of exchanging goods is 0, there is only one evolutionary stable equilibrium point (0, 0, 0) in the replication dynamic system, and pharmaceutical enterprises provide low-quality drugs at this time. When the cost of exchanging goods increases, the replication dynamic system is in an unstable state. As *T* further increases, the evolutionary stable equilibrium point of the replication dynamic system is (0, 1, 0), and pharmaceutical enterprises will consciously provide high-quality drugs in order to avoid additional expenditure on adjusting goods.

### Impact of additional cost

**Figure 8:**
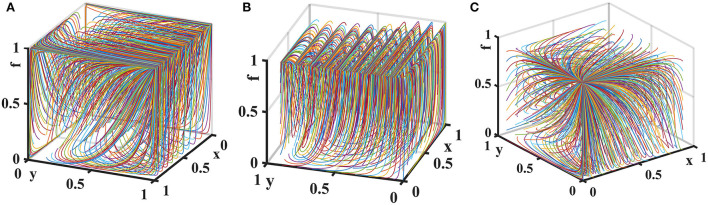
Impact of additional costs on the evolution of each player's strategy. Figure is the simulation diagram that shows the influence of the additional costs on the strategic choices of government regulator, pharmaceutical enterprises, and pharmaceutical e-commerce companies. **(A)** When *G*_*h*_−*G*_*l*_ = 0, *C*_*h*_−*C*_*l*_ = 0; **(B)** when *G*_*h*_−*G*_*l*_ = 3, *C*_*h*_−*C*_*l*_ = 3; **(C)** when *G*_*h*_−*G*_*l*_ = 7, *C*_*h*_−*C*_*l*_ = 7.

Let the probability that medical institutions choose inspection is *z* = 0.5. Consider the additional cost of a government regulator choosing strict supervision *G*_*h*_ − *G*_*l*_ = {0, 3, 7} and the additional cost for pharmaceutical enterprises to provide high-quality drugs *C*_*h*_ − *C*_*l*_ = {0, 3, 7}. The tripartite game evolutionary process and results of government regulator, pharmaceutical enterprises, and pharmaceutical e-commerce companies are shown in Figure 8.

It can be seen from Figure 8 that when the additional costs are 0, there is only one evolutionary stable equilibrium point (1, 1, 1) in the replication dynamic system. At this time, pharmaceutical enterprises provide high-quality drugs, government regulator strictly supervises, and pharmaceutical e-commerce companies choose inspection. As the additional costs increase, the replication dynamic system is in an unstable state. When additional costs further increase, the evolutionary stable equilibrium point of the replication dynamic system is (0, 0, 1). At this time, the government regulator loosely supervises, pharmaceutical enterprises provide low-quality drugs, and pharmaceutical e-commerce companies choose to inspect. Therefore, in drugs quality supervision of distribution channels in the pharmaceutical supply chain, fundamentally speaking, reducing the additional costs of each link can effectively improve the enthusiasm of all parties to choose positive behaviors and maintain a good market order.

## Discussions

This article considers the participants in different distribution channels of the pharmaceutical supply chain, and constructs a quartet evolutionary game model of government regulator, pharmaceutical enterprises, medical institutions, and pharmaceutical e-commerce companies. The stable equilibrium point of each strategic choice is solved, and the stability of the strategic combination of the replication dynamic system is analyzed. This article analyzes the influence of pharmaceutical enterprises' sales channels and various factors on the drugs quality of pharmaceutical supply chain and uses *MATLAB 2020b* simulation to verify. The main suggestions are as follows.

First of all, the amount of fines can effectively restrain the behavior of government regulator, pharmaceutical enterprises, medical institutions, and pharmaceutical e-commerce companies, but excessive punishment will inhibit economic behavior, easily lead to collusion, and is not conducive to maintaining a good market order. Therefore, it is necessary to establish a reasonable reward and punishment mechanism. Publicize and praise pharmaceutical enterprises, medical institutions, and pharmaceutical e-commerce companies that fulfill their social responsibilities, and set up a list of exemptions to encourage them.

Second, all parties, as rational subjects, pursue the maximization of interests. Only when their positive behavior can bring profits, they will prompt government regulator strictly supervise, pharmaceutical enterprises provide high-quality drugs, and medical institutions and pharmaceutical e-commerce companies inspect. Therefore, online platform and patient feedback mechanism can be introduced. Information sharing compensation mechanism can be designed to connect government regulator, pharmaceutical enterprises, medical institutions, pharmaceutical e-commerce companies, and other subjects, so that they can actively share drugs quality information and reduce information asymmetry and lag. At the same time, they can supervise each other and check and balance each other, which can reduce the additional cost of strict supervision by the government regulator and improve the supervision efficiency.

Eventually, the sales cost and sales price of different distribution channels are different. Compared with medical institutions, pharmaceutical e-commerce companies do not need to provide diagnosis and treatment services, and the sales cost is lower, which will weaken the advantages of offline medical institutions. In order to avoid monopoly and balance the market, pharmaceutical enterprises should differentiate wholesale prices between medical institutions and pharmaceutical e-commerce companies to maintain a stable business atmosphere.

## Conclusion

With the continuous development of Internet technology, pharmaceutical enterprises pay more and more attention to the innovation of supply chain services and the construction of core capabilities of professional pharmaceutical third-party logistics services. The competition between online pharmaceutical e-commerce companies and offline medical institutions is becoming increasingly fierce. Under the different distribution channel structures, the drugs quality supervision of the pharmaceutical supply chain is a common challenge faced by all countries in the world. Set up a reasonable reward and punishment mechanism for the main body of the pharmaceutical supply chain, encourage government regulator to strictly supervise, pharmaceutical enterprises to provide high-quality drugs, and medical institutions and pharmaceutical e-commerce companies to conduct the inspection. Introduce an online information platform to reduce information asymmetry, promote information sharing, and reduce the operating costs of supply chain members. Balancing the competition among different distribution channel entities and pricing reasonably, with the joint participation of all entities, can effectively guarantee the drugs quality level of the pharmaceutical supply chain.

Considering the strategic choices of government regulator, pharmaceutical enterprises, medical institutions, and pharmaceutical e-commerce companies, this article constructs an evolutionary game model for the drugs quality supervision of pharmaceutical supply chains under different distribution channels. However, the constructed game model is complete information under bounded rationality, one-stage, and does not consider the game order. Therefore, considering the influence of information asymmetry, building a multi-stage, repetitive and dynamic game model under the patient feedback mechanism is the next research direction.

## Data availability statement

The original contributions presented in the study are included in the article/supplementary material, further inquiries can be directed to the corresponding author/s.

## Author contributions

SZ wrote the manuscript, solved the models, and made data analysis. LZ designed the research question, constructed the models, and revised and edited the manuscript. All authors have read and approved the manuscript.

## Funding

This work was supported by the National Social Science Fund of China under grant Nos. 20BGL272 and 21ZDA024 and the Nature Science Foundation of Shandong Province in China under grant No. ZR2019MG017.

## Conflict of interest

The authors declare that the research was conducted in the absence of any commercial or financial relationships that could be construed as a potential conflict of interest.

## Publisher's note

All claims expressed in this article are solely those of the authors and do not necessarily represent those of their affiliated organizations, or those of the publisher, the editors and the reviewers. Any product that may be evaluated in this article, or claim that may be made by its manufacturer, is not guaranteed or endorsed by the publisher.
